# Transient Behavior and Control of Polyethylene Production in a Fluidized Bed Reactor Utilizing Population Balance Model

**DOI:** 10.3390/ijms25052602

**Published:** 2024-02-23

**Authors:** Nayef Ghasem

**Affiliations:** Department of Chemical & Petroleum Engineering, United Arab Emirates University (UAEU), Al-Ain P.O. Box 15551, United Arab Emirates; nayef@uaeu.ac.ae

**Keywords:** fluidized bed, polyethylene, polymerization, population balance, stability, PID control

## Abstract

In this study, a fluidized bed reactor for polyethylene production was employed using a dry mode approach, where the recycle stream may contain components of a nature that cannot be condensed through standard cooling. To analyze the behavior of the fluidized bed reactors during the copolymerization of ethylene with butene, a dynamic population balance model was employed. The study includes sensitivity analyses through computer simulations to examine the variations in reactor temperature, molecular weights, catalyst feed rate, and monomer/comonomer concentrations in the fluidized bed reactor. It is noteworthy that the reactor exhibits instability under normal operational conditions and is sensitive to changes in the catalyst feed rate and coolant temperature of the heat exchanger. The findings also highlight challenges such as temperature fluctuations above the polymer melting point. This underscores the importance of implementing a temperature control system to prevent issues like reactor shutdown due to elevated temperatures. Dynamic instabilities were observed under specific circumstances and were successfully controlled using Proportional Integral Derivative (PID) control strategies. The population balance model is essential for understanding the complexity of transient polymerization reactions. It enables researchers to simulate and optimize polymerization processes by utilizing the detailed kinetics of the reaction.

## 1. Introduction

Fluidized bed reactors exhibit fluid-like properties, rendering them sensitive to variations in temperature and pressure [1,2,3]. Safety is a crucial concern when operating any chemical reactor, including fluidized bed reactors utilized for polymerization processes [4]. It is essential to conduct a thorough analysis of the thermal behavior of these reactors under different conditions in respect to potential reactions and the formation of spots [5,6]. Moreover, the polymer industry strives for more sustainable practices to explore ways to enhance energy efficiency in fluidized bed reactors [7]. By optimizing their stability, researches are seeking to reduce energy consumption, minimize greenhouse gas emissions, and contribute to environmentally friendly ethylene polymerization processes [8]. 

In recent years, there has been a significant increase in the demand for polyethylene within the polymer industry [9,10,11]. This is primarily due to its versatile applications in packaging, construction, and various other sectors. Polyethylene (PE) is a commonly used resin, and it can be classified into several types based on its density, such as high-density polyethylene (HDPE), medium-density polyethylene (MDPE), and low-density polyethylene (LDPE). Among the technologies employed for ethylene polymerization are fluidized bed reactors that offer advantages such as better control of overheating and mass transfer under reaction conditions, while being scalable. It is worth mentioning that ensuring the stability of these reactors plays a vital role in the successful polymerization process [12,13,14,15]. In ethylene polymerization within a fluidized bed reactor, the condensed mode is one approach. This method considers factors like understanding multiphase flow characteristics, heat transfer behaviors in gas-phase fluidized beds, and catalyst reaction kinetics [16,17,18]. The goal was to boost the capacity of traditional ethylene polymerization reactors. Surprisingly, the industrialization of condensed mode operations in gas-phase fluidized beds challenged the belief that recycled gas could not carry liquid during ethylene polymerization in such beds. When ethylene polymerization is conducted using fluidized bed reactors in a condensed mode operation, the liquid condensate plays a role in the polymerization process [19,20]. It helps absorb and remove heat from the flowing recycle stream through evaporation. This significantly enhances the heat removal capability of the fluidized flow. At the time, condensate components like isopentane and 1-hexene are absorbed by polyethylene particles, causing them to swell. This swelling both improves the ethylene diffusion coefficient, within mesoscale and microscale pores, and enhances the phase of polyethylene particles [21]. As a result, introducing condensate into the process leads to an acceleration in the rate of ethylene polymerization, enhancing the production capacity of the fluidized bed. When the evaporation time of the liquid and the circulation time of polyethylene particles in the fluidized bed are synchronized, specific reaction conditions are formed in localized zones. These zones have a hydrogen/ethylene ratio and a high copolymer monomer (such as 1-butene 1-hexene)/ethylene ratio. The concentration of copolymers and absence of a chain transfer agent like hydrogen promote the growth of copolymer monomers into molecular weight branched chains, thereby improving the properties of the product [22,23,24,25]. Condensate has an impact on both stability and thermal stability in fluidized bed reactors. This impact affects particle aggregation and fluidization behaviors. Fluidizing dynamics models have been developed to study how liquid vaporization affects fluidized bed temperature, particle growth rate, and hydrodynamic behaviors. Their findings suggest that evaporating condensate alters both gas velocity distribution and flow structure within the fluidized bed [26,27,28]. This transformation creates a polymerization reaction environment with phases and zones within the fluidized bed, providing opportunities to customize the chain structures of polyethylene [29]. Different strategies have been proposed to adjust feeding parameters, like hydrogen, monomers, and copolymer monomers to synthesize polyethylene with a desired molecular weight distribution [30]. However, adjusting the hydrogen feeding rate to regulate the chain structure of polyethylene is crucial. Some research has also explored the operation of polymerization to regulate the chain structure of polyethylene [31,32,33]. By addressing concerns related to stability, we do not only ensure the consistent production of high-quality polyethylene but also contribute to the economic viability and environmental sustainability of the polymerization process [34,35,36]. By contrast, condensed mode operation can lead to the fastest catalyst deactivation; the harsh conditions in the condensed mode can accelerate equipment wear and tear [37].

Building on these technologies, this research focuses on studying dry dynamics behavior, considering the population balance model that includes the balance of the inactive, potential active catalyst sites, first and second moments of live and dead polymers, along with temperature, in the stability of fluidized bed reactors used for ethylene polymerization. The model investigates the influence of operating parameters, like gas feed concentrations, catalyst feed rate, and temperature, on the behavior of the system, mainly the polyethylene molecular weight. The goal is to comprehend the challenges associated with utilizing fluidized bed reactors for ethylene polymerization and to explore the regulation of state variables within these reactors. The reactor was stabilized by employing a PID control approach. Apart from its impact on designing and ensuring safety in reactors, the findings of this research could significantly influence the way polymer manufacturing is conducted in the future. It aims to align the industry with the goals of efficiency, safety, and environmental responsibility.

## 2. Results and Discussion

### 2.1. Significance of Controlling Unstable Systems 

It is essential to maintain control over unstable systems to prioritize safety, reliability, and efficiency in a range of industries. Unpredictable issues within these systems can result in accidents and disruptions, emphasizing the importance of implementing control mechanisms. By achieving stability, we not only ensure performance and compliance with regulations but also enable progress and innovation across sectors such as aerospace and manufacturing. Through the regulation of variables and the minimization of impact, controlling unstable systems contributes to overall operational success and the well-being of society.

In ethylene polymerization in fluidized bed reactors, the speed at which the catalyst is added (qc) is a significant factor that affects the polymerization process. The catalyst plays a role in initiating and regulating the reactions that lead to polymerization within the reactor. The specific feed rate at which the catalyst is added can vary depending on factors such as reactor design, polymerization conditions, and the desired characteristics of the polyethylene product. Finding the optimal catalyst feed rate is essential in achieving the desired polymerization rate, molecular weight distribution, and other properties of the product. This optimization process involves process refinement, considering variables like reactor temperature, pressure, monomer feed rate, and other relevant parameters. 

**Figure 1:**
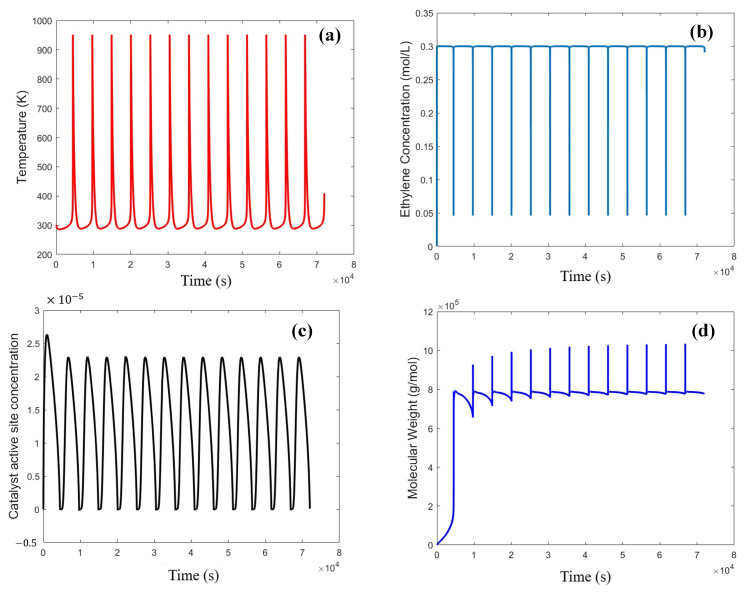
Reactor behavior under open-loop conditions; reactor exit temperature (**a**), ethylene concentration (**b**), catalyst active site concentration (**c**), and polyethylene molecular weight (**d**) are observed over time during an open-loop operation under fixed feed conditions (ethylene 0.3 mol/L, butene 0.1 mol/L, hydrogen 0.02 mol/L, catalyst speed rate 0.2 g/s).

In Figure 1, the temperature variations (Figure 1a), catalyst potential active site, catalyst active site, ethylene concentration, and polyethylene molecular weight are illustrated during open-loop operations under conventional fluidized bed operating conditions. The figure distinctly highlights the system’s instability under these conditions, emphasizing the necessity for a control scheme to stabilize the system.

### 2.2. Effect of Catalyst Speed Rate

**Figure 2:**
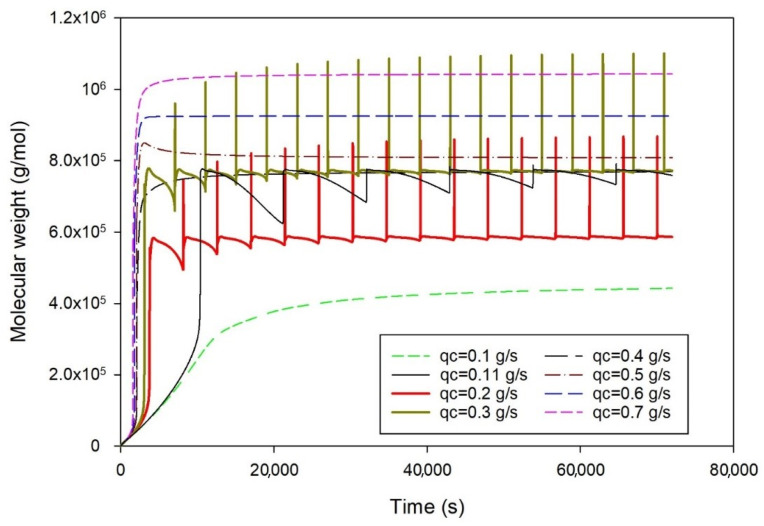
The variation of polyethylene molecular weight with time at fixed other conditions (ethylene 0.3 mol/L, butene 0.1 mol/L, hydrogen 0.02 mol/L), and different catalyst feed rates, qc (0.1 to 0.7 g/s); heat exchanger cooling temperature is fixed at 283 K.

Figure 2 depicts the effect of the catalyst feed rate (0.1 to 0.7 g/s) on polymer molecular weight with time. The figure reveals that an increase in the catalyst feed rate leads to a higher rate of polymerization, resulting in the production of longer polymer chains and higher-molecular-weight polyethylene. The presence of more catalysts in the reactor provides additional active sites for polymerization. This is attributed to the fact that a higher catalyst concentration results in more active sites being available for the polymerization process. At catalyst feed rates of 0.11, 0.2, and 0.3 g/s, the figure illustrates unstable behavior, indicating a range of catalyst feed rates where the systems fall into instability [17]. After a thorough simulation, this unstable region was identified to be between catalyst feed rates of 0.11 and 0.38 g/s. 

### 2.3. Effect of Ethylene Concentrations

The level of ethylene concentration in a fluidized bed reactor has an impact on the production of polyethylene. The concentration of ethylene plays a role in determining how quickly the polymerization process occurs, with higher concentrations resulting in higher production rates. It also affects the molecular weight distribution of the polyethylene, which in turn influences properties like density melt flow rate and crystallinity. Managing the stability of the fluidized bed reactor is also affected by ethylene concentration; it needs to be kept at levels to avoid problems such as fouling or shutdown. Moreover, adjusting the ethylene concentration allows for the customization of polymer properties to meet application requirements, impacting factors like energy consumption during polymerization and overall economic viability [12]. 

**Figure 3:**
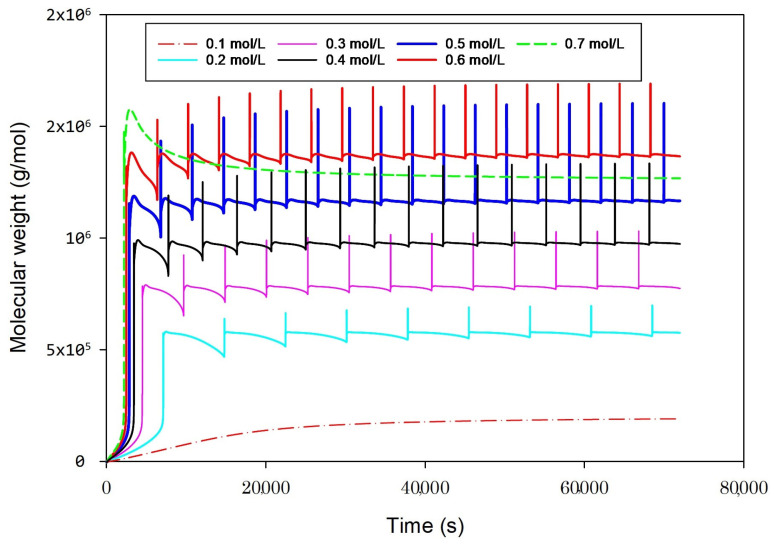
Effect of ethylene concentrations (0.3 mol/L, 0.5 mol/L, 0.7 mol/L) on molecular weight distribution at fixed other conditions, qc = 0.2 g/s, butene 0.1 mol/L, hydrogen 0.02 mol/L, heat exchanger cooling temperature 283 K.

Figure 3 describes the effect of ethylene feed concentration on polymer molecular weight; it reveals that an increase in ethylene feed concentrations leads to longer polymer chains and a higher molecular weight. That is attributed to the abundance of ethylene, which reduces the likelihood of chain transfer reactions during the polymerization process. The system exhibits increased stability as the ethylene concentration rises, facilitating the production of high-molecular-weight polyethylene. At ethylene feed concentrations of 0.2, 0.3, 0.4, 0.5, and 0.6 mol/L, the system exhibits unstable behavior with severe oscillations. As the ethylene concentration is increased to 0.7 mol/L, the system is prone to a stable state where the oscillations vanish. 

### 2.4. Effect of Butene (Comonomer) Feed Concentrations

The amount of butene used as a comonomer in fluidized bed reactors for ethylene polymerization plays a role in determining the production and molecular weight distribution of polyethylene. When butene concentrations are increased, it affects the rate at which it copolymerizes with ethylene, resulting in reaction kinetics and the formation of copolymers with varying properties. These actions directly impact the distribution of molecular weights in the produced polyethylene, leading to variations in the polymer chain lengths. Furthermore, the presence of butene in the polymer structure has a significant impact on its mechanical properties and overall structure. The extent of branching and distribution of butene along the polymer chain is determined by its concentration. By adjusting these concentrations, it becomes possible to tailor the properties of polymers, such as crystallinity, thermal stability, and melt flow characteristics, to meet specific application requirements. Maintaining appropriate concentration levels is crucial for the smooth functioning of fluidized bed reactors as it affects their efficiency, potential fouling issues, and overall stability. In summary, precise control over concentrations is vital not only for achieving the desired characteristics in polyethylene production but also for ensuring consistent performance in fluidized bed operations [38].

**Figure 4:**
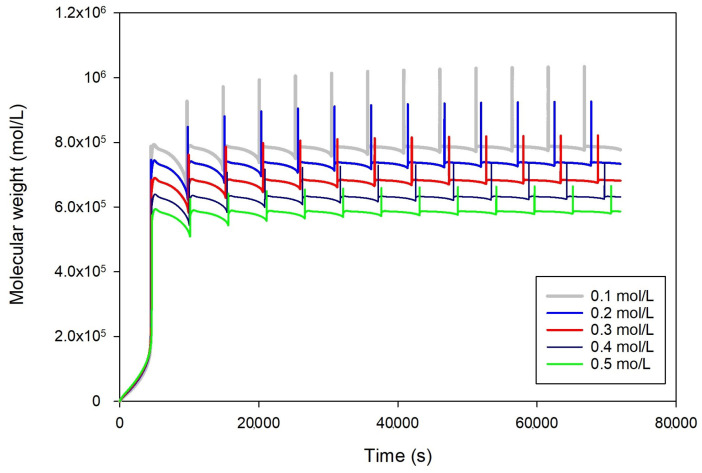
Effect of butene feed concentrations (0.1 to 0.5 mol/L) on molecular weight distribution at fixed other conditions, qc = 0.2 g/s, ethylene 0.3 mol/L, hydrogen 0.02 mol/L, heat exchanger cooling temperature 283 K.

The effect of butene comonomer feed concentrations on system performance and molecular weight is represented in Figure 4. The figure reveals that the presence of butene as a commoner impacts the molecular weights of the formed polyethylene. When there are high concentrations of butene, it increases polymer chains and lowers molecular weights; as the amount of butene increases, there is a chance of branching occurring in the polymer chains. This increase in branching results in a decrease in the crystallinity of the polymer. In general, increased concentrations of butene can result in the formation of polymer chains and lower molecular weights, because of chain transfer reactions. The oscillation appears in the five butene feed concentrations (0.1, 0.2, 0.3, 0.4, and 0.5 mol/L). By contrast, the oscillation altitude declines with increased butene concentrations. 

### 2.5. Effect of Hydrogen Feed Concentration

The rate at which hydrogen is fed into the fluidized bed reactor has an impact on both the polyethylene production rate and the polyethylene molecular weight. This factor plays a role in determining how fast the polymerization process occurs in the reactor, as higher hydrogen feed rates lead to increased polyethylene production rates. Additionally, the rate at which hydrogen is fed into the reactor is a factor in controlling the average molecular weight of the resulting polyethylene. It has an impact on the growth of polymer chains during polymerization and ultimately shapes the range of weights. Adjusting the rate at which hydrogen is fed also allows for fine-tuning polyethylene properties, such as density, melt flow rate, and crystallinity. The stability of the fluidized bed reactor heavily relies on controlling the hydrogen feed rate to prevent issues like fouling and to ensure consistent polymerization rates. Additionally, variations in the hydrogen feed rate directly influence the distribution of polymer chain lengths, thereby affecting the polyethylene’s properties. From an optimization perspective, finding the right balance in setting the inlet hydrogen feed rate involves considering the desired polymer properties and cost-effectiveness factors like hydrogen’s expense and the overall process efficiency [39]. Essentially, it is crucial to regulate the rate at which hydrogen is supplied to the reactor’s inlet. This is necessary to obtain the desired polyethylene properties and ensure the effectiveness of the polymerization process taking place in the fluidized bed reactor. During the process of ethylene polymerization in a fluidized bed reactor, the molecular weight of polyethylene can be influenced by factors including the concentration of hydrogen in the feed. To control the molecular weight of the polymer, hydrogen is commonly used as a chain transfer agent in polyethylene polymerization reactions. 

**Figure 5:**
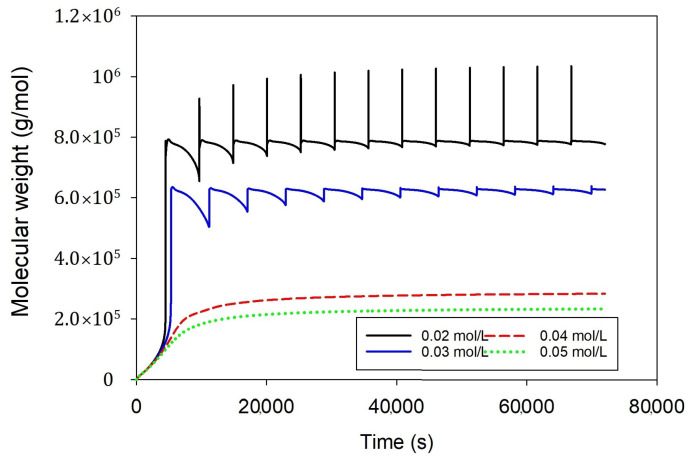
Effect of hydrogen feed concentrations (0.02, 0.03, 0.04, and 0.05 mol/L) on molecular weight at fixed other conditions, qc = 0.2 g/s, ethylene 0.3 mol/L, butene 0.1 mol/L, heat exchanger cooling temperature 283 K.

Figure 5 represents the influence of hydrogen concentration on polyethylene molecular weight. Increasing the hydrogen concentration promotes chain transfer reactions, resulting in shorter polymer chains and lower molecular weights. Higher concentrations of hydrogen generally lead to an increased rate of polymerization and a decrease in the molecular weight of the produced polyethylene. Conversely, reducing the amount of hydrogen decreases chain transfer reactions, allowing for long polymer chains and higher molecular weights. Lower levels of hydrogen concentration can decelerate the pace of polymerization, promoting the formation of polyethylene with higher molecular weights. It should be noted that a hydrogen concentration of 0.02 mol/L and 0.03 mol/L might render the system unstable. Conversely, when utilizing higher hydrogen feed concentrations (0.04, 0.05 mol/L), the reactor achieves stability; however, it results in a decrease in polyethylene molecular weight.

### 2.6. Effect of Gas Inlet Temperature 

The temperature of the feed gas inside a fluidized bed reactor plays a vital role in determining the production of polyethylene, its molecular weight distribution, and the stability of the system. This temperature directly affects how quickly polymerization occurs; higher feed temperatures lead to faster polymerization reactions and, as a result, higher rates of polyethylene production. Additionally, the temperature of the feed gas controlled by the coolant temperature is a crucial factor in controlling the molecular weight of the produced polyethylene. It influences how polymer chains grow and shapes the overall distribution of molecular weights. By adjusting the temperature of the feed gas, we can fine-tune various properties of polyethylene such as density, melt flow rate, and crystallinity. Maintaining stable conditions within the fluidized bed reactor relies heavily on precise control over both the feed gas and recycled gas temperatures. This helps prevent issues like fouling and ensures consistent rates of polymerization. Managing the feed gas temperature also plays a significant role in optimizing energy efficiency throughout the polymerization process. In summary, maintaining an optimal feed gas temperature is essential for achieving the desired characteristics in polyethylene, controlling molecular weight variations, and ensuring stable and efficient operation within the fluidized bed reactor. The combination of the feed gas and recycled gas temperatures is influenced by factors such as the coolant water temperature and flow rate in the heat exchanger [40].

**Figure 6:**
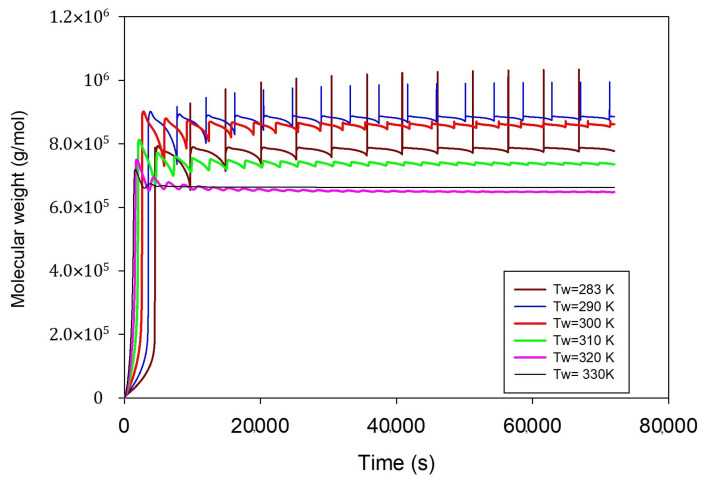
Effect of cooling water temperature on polyethylene molecular weight at fixed other conditions, qc = 0.2 g/s, ethylene 0.3 mol/L, butene 0.1 mol/L, hydrogen 0.02 mol/L.

According to Figure 6, the temperature inside the reactor is a factor that determines the desired molecular weight of polyethylene. When the water-cooling temperature in the heat exchanger is low, it leads to a higher rate of heat removal and consequently lower reactor temperatures. This can potentially impact the reaction kinetics and polymerization rate. By contrast, a higher coolant temperature can help in maintaining the reactor temperature desired for specific polymerization conditions. In general, when the reactor temperature is low, it tends to result in higher molecular weights, because it reduces the occurrence of chain termination and chain transfer reactions. On the other hand, maintaining higher cooling temperatures can help keep the reactor temperature high, which is beneficial for producing polyethylene with a higher molecular weight. However, attention should be paid to avoid polymer overheating that may result in polymer melting and, consequently, reactor shutdown. Figure 6 reveals that the system is prone to unstable situations for coolant temperatures between 283 to 320; by contrast, the system gains stability for coolant temperatures of 330 K. 

### 2.7. Molecular Weight Distribution

**Figure 7:**
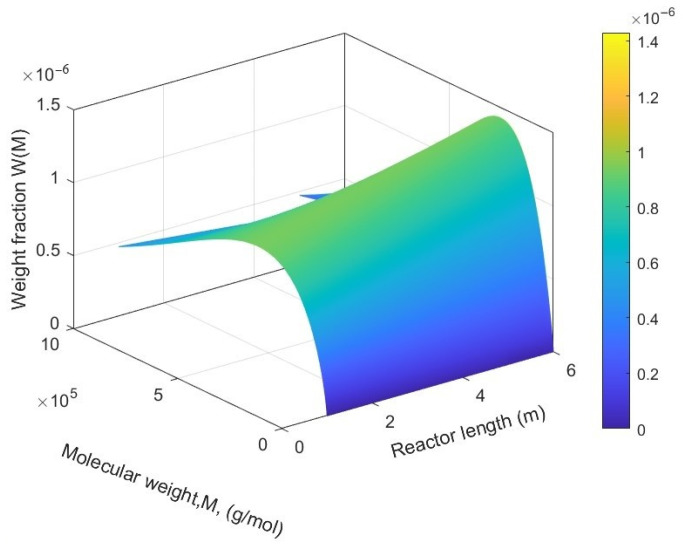
Molecular weight distribution (MWD) along the length of the fluidized bed reactor; catalyst feed rate is 0.3 g/s.

Molecular weight distribution (MWD) refers to the range of molecular weights available in a sample of polymer and the relative abundance of each molecular weight within that range. It describes the distribution of polymer chain lengths within a polymer product. The distribution of molecular weights plays a role in determining the characteristics and effectiveness of polymers across different applications. For instance, if the molecular weight distribution is more consistent, it can result in materials with consistent properties. On the other hand, a broader distribution can impact factors like strength, viscosity, and how easy it is to process the polymer. Scientists often employ methods like gel permeation chromatography (GPC) or size exclusion chromatography (SEC) to assess the molecular weight distribution of polymers. Figure 7 illustrates molecular weight distribution along the length of the reactor. 

### 2.8. Influence of PID Control on System Dynamics

**Figure 8:**
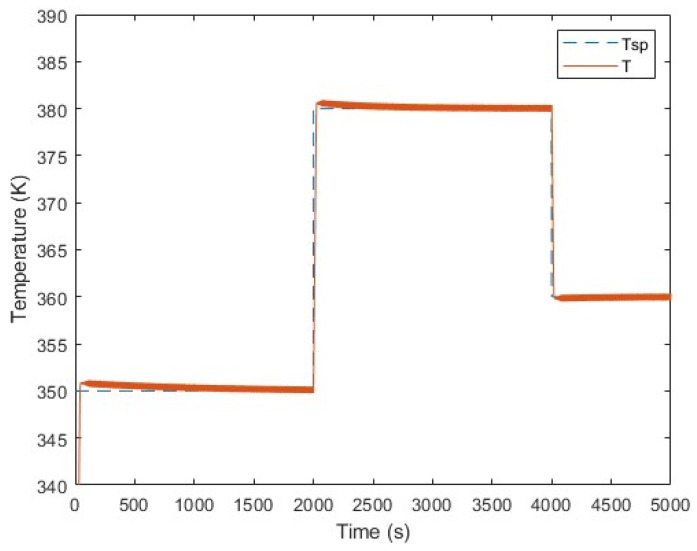
Regulating temperature through various incremental steps (set point tracking), maintaining a qc of 0.2 g/s, with ethylene at 0.3 mol/L, butene at 0.1 mol/L, and hydrogen at 0.02 mol/L.

Proportional Integral Derivative (PID) control methods have a significant impact on the performance of polyethylene fluidized bed reactors. These control systems play a role in maintaining temperature control within the reactor. This directly affects the reaction rates of polymerization, as well as the properties of the resulting polyethylene, such as molecular weight. By adjusting the rates at which monomer is supplied, PID controllers play a crucial role in controlling the distribution of molecular weight, thus influencing the characteristics of the final product. Another important function of these control strategies is to ensure stability in the reactor by addressing issues like temperature fluctuations, fouling, and unexpected shutdowns. Moreover, PI and PID controllers help optimize polyethylene production rates by adjusting feed rates to meet desired levels, while maintaining product quality and reactor stability. Additionally, these control strategies also contribute to improving energy efficiency by optimizing the use of cooling systems and heaters [41]. The incorporation of a component in PID controllers allows for responsiveness to disturbances, enabling quick system recovery and stable operation even in dynamic environments. In summary, implementing PID control methods is crucial for achieving control, stability, and efficiency in polyethylene fluidized bed reactors. Figure 8 illustrates how reactor temperature is regulated in response to changes in the temperature set point. The figure demonstrates that the PID control effectively manages to regulate the reactor temperature and tracks the step changes in the set point (Tsp) under various conditions, while minimizing temperature (T) overshooting. Figure 8 demonstrates the performance of a robust controller that tracks the change in the reactor temperature set point successfully [42].

Several factors throughout the polymerization process are influenced by adjusting the concentration of monomers such as ethylene, which can affect the length of polymer chains. Higher concentrations typically lead to longer chains and higher molecular weights. Introducing comonomers like butene allows us to introduce variations in the polymer structure, which influence branching and tacticity, thereby enabling us to control molecular weight. The presence of hydrogen as a chain transfer agent gives us further control over molecular weight by adjusting its concentration. The choice of catalyst type and feed rate also plays a significant role, since catalysts can be tailored to produce polymers with specific molecular weights. Reaction conditions, including temperature and pressure, impact the kinetics of polymerization and consequently affect weight as well. Additionally, factors such as reaction time, residence time, and the use of inhibitors or stabilizers all contribute to achieving precise control of molecular weight [43,44,45].

**Figure 9:**
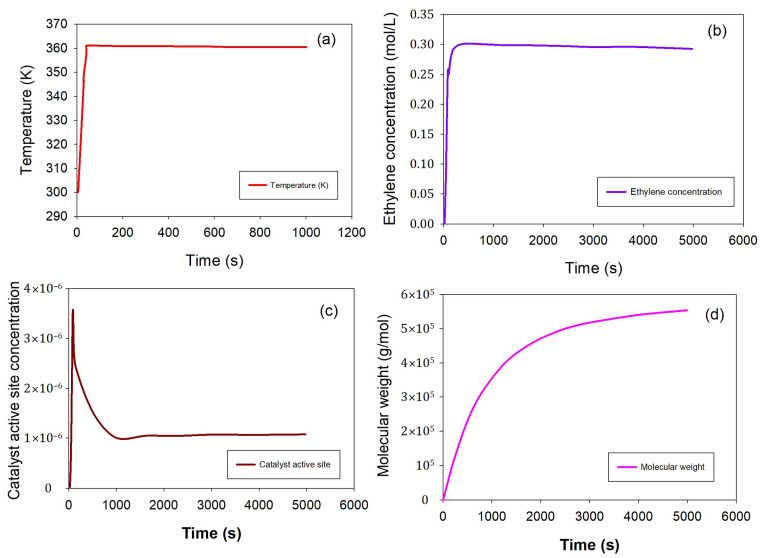
Under PID temperature control, the system has achieved stabilization at a set point of 365 K. This stability is depicted through various aspects: (**a**) temperature profile, (**b**) ethylene concentration, (**c**) catalyst active site, and (**d**) polyethylene molecular weight. These observations are made under fixed inlet conditions, with qc set at 0.2 g/s, ethylene at 0.3 mol/L, butene at 0.1 mol/L, and hydrogen at 0.02 mol/L.

Temperature variations inside the reactor have an impact on the polymer molecular weight due to the PID temperature control. This precision in controlling polyethylene molecular weight is crucial for tailoring polymers with predictable characteristics that meet the specific needs of various industries such as the packaging, automotive manufacturing, and medical fields. In contrast to Figure 1, Figure 9 illustrates the influence of maintaining a temperature control set point at 360 K on a range of parameters: (a) reactor exit temperature, (b) ethylene concentration, (c) catalyst active site, and (d) polymer molecular weight. The molecular weight exhibits a continuous increase at the controlled temperature set point, without any observed oscillations. This absence of oscillations indicates that the system is predisposed to stable operation, implying that it sustains a consistent and controlled behavior without significant fluctuations. This stability is a crucial characteristic, ensuring the reliability and predictability of the overall manufacturing process under the specified conditions.

## 3. Material and Methods

### 3.1. Model Equations

**Figure 10:**
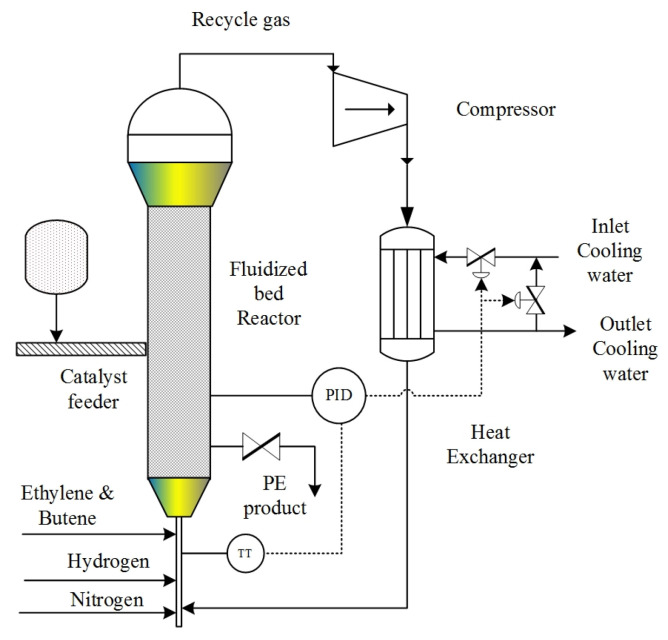
Schematic diagram of ethylene polymerization in fluidized bed reactor. Diameter 0.5 m, height 5 m. The inlet arrows represent the feed streams, and the exit arrows represent the outlet streams.

The diagram in Figure 10 illustrates the system of ethylene polymerization, incorporating a fluidized bed reactor. The dynamic mathematical model developed to describe the micro material and energy balance of the fluidized bed reactor for polyethylene production takes into consideration the following assumptions [28].

1. Neglecting radial gradient and axial dispersion (plug flow assumption).
2. Neglecting the heat effect due to activation, initiation, and chain transfer.
3. The polymerization reaction takes place on a dense solid phase.
4. Neglecting the change in bed void fraction, gas, and solid velocities along the bed.

The kinetic constants required to solve the population balance model equations are shown below, and the related reaction mechanism can be found elsewhere [28].

$$kn(1/s)=7.19557{\times 10}^{4}e^{\left(-\frac{8000}{Rg.T}\right)}$$

The units of the following rate constants are L/(mol.s)

$$ki1={2.91205\times 10}^{5}e^{\left(-\frac{9000}{Rg.T}\right)}$$

$$ki2=4.07687\times 10^{4}e^{\left(-\frac{9000}{Rg.T}\right)}$$

$$kh1=6.33210\times 10^{3}e^{\left(-\frac{9000}{Rg.T}\right)}$$

$$kh2=6.33210\times 10^{3}e^{\left(-\frac{8000}{Rg.T}\right)}$$

$$kp11=2.47524\times 10^{3}e^{\left(-\frac{9000}{Rg.T}\right)}$$

$$kp12=5.82409\times 10^{5}e^{\left(-\frac{9000}{Rg.T}\right)}$$

$$kp21=1.86371\times 10^{7}e^{\left(-\frac{9000}{Rg.T}\right)}$$

$$kp22=4.36807\times 10^{5}e^{\left(-\frac{9000}{Rg.T}\right)}$$

$$kf11=1.51107\times 10^{2}e^{\left(-\frac{8000}{Rg.T}\right)}$$

$$kf12=4.31734\times 10^{2}e^{\left(-\frac{8000}{Rg.T}\right)}$$

$$kf21=1.51107\times 10^{2}e^{\left(-\frac{8000}{Rg.T}\right)}$$

$$kf22=4.31734\times 10^{2}e^{\left(-\frac{8000}{Rg.T}\right)}$$

Pseudo rate constants

$$ki=(M_{1}\times ki1+M_{2}\times ki2)/M$$

$$kp1=(M_{1}\times kp21\times kp11+M_{2}\times kp12\times kp21)/(M_{1}\times kp21+M_{2}\times kp12)$$

$$kp2=(M_{1}\times kp21\times kp12+M_{2}\times kp12\times kp22)/(M_{1}\times kp21+M_{2}\times kp12)$$

$$kp=(M_{1}\times kp1+M2\times kp2)/M$$

$$kf1=(M_{1}\times kp21\times kf11+M_{2}\times kp12\times kf21)/(M_{1}\times kp21+M_{2}\times kp12)$$

$$kf2=(M_{1}\times kp21\times kf12+M_{2}\times kp12\times kf22)/(M_{1}\times kp21+M_{2}\times kp12)$$

$$kf=(M_{1}\times kf1+M_{2}\times kf2)/M$$

$$kh=(M_{1}\times kp21\times kh1+M_{2}\times kp12\times kh2)/(M_{1}\times kp21+M_{2}\times kp12)$$

### 3.2. Model Partial Differential Equations

The balance on the inactive catalyst site, the potential active site (*R*):

$$\frac{\partial R}{\partial t}=v_{solid}\frac{\partial R}{\partial z}-kn\times R$$

The catalyst active site balance $P_{0}$:

$$\frac{\partial P_{0}}{\partial t}=v_{solid}\;\frac{\partial P_{0}}{\partial z}+kn\times R-ki\;{\times P}_{0}\times M$$

The hydrogen material balance

$$\frac{\partial H_{2}}{\partial t}=v_{gas}\;\;\;\frac{\partial H_{2}}{\partial z}\;-kh\times mu0\times H_{2}\;\frac{\left(1-void\right)}{void}$$

where *phi* = 1 − *void*, and void indicates the void fraction of the fluidized bed.

The ethylene (*M*_1_) material balance

$$\frac{\partial M_{1}}{\partial t}=v_{gas}\frac{\partial M_{1}}{\partial z}-\left(ki1\times P_{0}+kp1\times mu0+kf1\times mu0\right)\times M_{1}\;\frac{\left(1-void\right)}{void}$$

The butene (*M_2_*) material balance

$$\frac{\partial M_{2}}{\partial t}=v_{gas}\;\;\frac{\partial M_{2}}{\partial z}-\left(ki2\times P_{0}+kp2\;.mu0+kf2\times mu0\right)\times M_{1}\frac{\left(1-void\right)}{void}$$

Polymer characterization based on live moment (mu0, mu1, mu2), zero, first, and second, respectively.

$$\frac{\partial mu0}{\partial t}=v_{solid}\frac{\partial mu0}{\partial z}+ki\times P_{0}\times M-kh\times mu0\times H_{2}$$

$$\frac{\partial mu1}{\partial t}=v_{solid}\frac{\partial mu1}{\partial z}+ki\times P_{0}\times M-kh\times mu1\times H_{2}+kp\times mu0\times M+kf\times \left(mu0-mu1\right)\times M$$

$$\frac{\partial mu2}{\partial t}=v_{solid}\;\frac{\partial mu2}{\partial z}+ki\times P_{0}\times M-kh\times mu2\times H_{2}+kp\times \left(2mu1+mu0\right)\times M+kf\;\left(mu0-mu2\right)\times M$$

Polymer characterization based on dead moment (nu0, nu1, nu2), zero, first, and second, respectively.

$$\frac{dnu0}{dt}=v_{solid}\;\frac{\partial nu0}{\partial z}+kf\times mu0\times M+kh\times mu0\times H_{2}$$

$$\frac{dnu1}{dt}=v_{solid}\;\;\frac{\partial nu1}{\partial z}+kf\times mu1\times M+kh\times mu1\times H_{2}$$

$$\frac{dnu2}{dt}=v_{solid}\frac{\partial {nu}_{2}}{\partial z}+kf\times \;mu2\times M+kh\times mu2\times H_{2}$$

The reactor energy balance

$$\frac{dT}{dt}=\left(void\times v_{gas}\left(M+N_{2}+H_{2}\right)Cp_{g}+phi\times v_{solid}\times rhop\times Cp_{poly}\right)\frac{\partial T}{\partial z}+DeltaH\times kp\times M\times mu0\times phi)/(void\left(M+N_{2}+H_{2}\right)Cp_{g}+phi\times rhop\times Cp_{poly})$$

The average gas heat capacity ($Cp_{g}$)

$$Cp_{g}=(CpM_{1}\times M_{1}+CpM_{2}\times M_{2}+CpH_{2}\times H_{2}+CpN_{2}\times N_{2})/(M_{1}+M_{2}+H_{2}+N_{2})$$

*M* is the sum of concentrations of ethylene (*M*_1_) and butene (*M*_2_) in (mol/liter):

$$M=M_{1}+M_{2}$$

Gas ($v_{gas}$) and solid ($v_{solid}$) velocities (m/s), using the Haider–Levenspiel equation.

$$v_{solid}=v_{gas}-\frac{\mu_{g}}{\rho_{g}d_{p}}\frac{Ar}{18+0.591Ar^{\frac{1}{2}}}$$

The parameters used in solving the model are listed in Table 1.

The number of average molecular weight ($M_{n}$)

$$M_{n}=\left(\frac{{mu}_{1}+{nu}_{1}}{{mu}_{0}+{nu}_{o}}\right)\frac{{Mw}_{1}\times M_{1}+{Mw}_{2}\times M_{2}}{M_{1}+M_{2}}$$

The weight average molecular weight ($M_{w}$)

$$M_{w}=\left(\frac{{mu}_{2}+{nu}_{2}}{{mu}_{1}+{nu}_{1}}\right)\frac{{Mw}_{1}{\times M}_{1}+{Mw}_{2}{\times M}_{2}}{M_{1}+M_{2}}$$

where $Mw_{1}$, $Mw_{2}$, are the molecular weights of ethylene (monomer) and butene (comonomer).

The model equations were solved using the MATLAB/SIMULINK software package version 2023.

## 4. Conclusions

The process of polymerizing ethylene in a fluidized bed reactor is quite complicated and influenced by several factors. These factors include the concentration of hydrogen, comonomers like butene, the concentration of ethylene feed rate, catalyst feed rate, and the cooling temperature at which water is used for cooling. The concentration of hydrogen acts as a regulator for molecular weight, influencing chain transfer reactions and leading to polyethylene with varying molecular weights. Comonomers like butene introduce branching into polymer chains, which affects the molecular weight, tacticity, and other properties of the polymer. The concentration of ethylene in the feed has an impact on both the polymerization rates and the distribution of molecular weights. Higher concentrations generally lead to polymer chains with higher molecular weights. The rate at which the catalyst is introduced into the reactor influences both the speed of polymerization and overall efficiency. Higher catalyst feed rates often result in faster polymerization rates and longer chains in the produced polyethylene. However, it is important to control this rate to avoid issues such as catalyst deactivation and reactor instability. Additionally, maintaining a stable temperature through water cooling in a heat exchanger is critical. The cooling temperature directly affects heat removal capacity, which controls reaction kinetics and subsequently impacts the polyethylene molecular weight. Efficient implementation of Proportional Integral Derivative (PID) control strategies in fluidized bed reactors for polyethylene production is vital for optimizing various aspects of the polymerization process and stabilizing system variables like temperature and molecular weight. These control systems have a direct influence on temperature regulation, which in turn affects polymerization rates and ultimately shapes the properties of the resulting polyethylene. PID controllers play a significant role in refining molecular weight distribution and ultimately impacting the characteristics of the final product by adjusting variables such as monomer feed rates.

## Figures and Tables

**Table 1:** Parameters used in solving model equations [28].

Parameter	Value	Definition (Unit)
Rg	1.9871	Gas constant (Cal/mol K)
DeltaH	9.45 × 10^4^	Heat of polymerization (J/mol)
CpM1	46.02	Ethylene heat capacity (J/mol K)
CpM2	100.416	Butene heat capacity (J/mol K)
CpH2	27.6144	Hydrogen heat capacity (J/mol K)
CpN2	27.196	Nitrogen heat capacity (J/mol K)
N2	0.64	Nitrogen concentration mol/liter
rhop	950	Polymer density (g/liter)

## Data Availability

Data are contained within the article.

## Nomenclature

Ar	Archimedes number
H_2_	Hydrogen concentration
*M*_1_	Ethylene concentration
*M*_2_	Butene concentration
*mu*0	Zero live moment
*mu*1	First live moment
*mu*2	Second live moment
*nu*0	Zero dead moment
*nu*1	First dead moment
*nu*2	Second dead moment
*P*_0_	Active sites
*R*	Potential active sites
t	Integration time
T	Reactor temperature
